# Multiferroic Phases and Transitions in Ferroelectric Lead Titanate Nanodots

**DOI:** 10.1038/srep45373

**Published:** 2017-04-03

**Authors:** Tao Xu, Takahiro Shimada, Yoshitaka Uratani, Xiaoyuan Wang, Jie Wang, Takayuki Kitamura

**Affiliations:** 1 Department of Mechanical Engineering and Science, Kyoto University, Nishikyo-ku, Kyoto 615-8540, Japan; 2 Institute of Systems Engineering, China Academy of Engineering Physics, Postbox 919-401, Mianyang 621900, China; 3 Department of Engineering Mechanics, School of Aeronautics and Astronautics, Zhejiang University, Hangzhou 310027, China

## Abstract

Discovery of novel phases and their associated transitions in low-dimensional nanoscale systems is of central interest as the origin of emergent phenomena and new device paradigms. Although typical ferroelectrics such as PbTiO_3_ exhibit diverse phase transition sequences, the conventional incompatible mechanisms of ferroelectricity and magnetism keep them as simply nonmagnetic phases, despite the immense practical prospective of multiferroics in novel functional devices. Here, we demonstrate using density function theory that PbTiO_3_ nanodots exhibit unconventional multiferroic phase transitions. The nanosize and nonstoichiometric effects intrinsic to nanodots bring about the coexistence of ferromagnetism with the host electric polarization, mediated by the termination and surface morphology. We also predict the key features of the size-dependent phase diagram of nanodots that involve a rich sequence of ferroelectric-multiferroic-ferromagnetic/nonmagnetic (FE-MF-FM/NM) multiferroic phase transitions. The present work thus provides an avenue to realizing multiferroics and multifunctional oxides in low-dimensional systems.

The pursuit of unusual phases and their correlated transitions is at the very core of condensed matter science. In particular, discovering the phase transitions in ferroic materials have remained a primary goal in this endeavor due to the rich physical phenomena and tremendous technological promise^1^,^2^,^3^. Multiferroic phases with the coexistence and cross-coupling of different ferroic orders such as ferroelectricity and ferromagnetism^4^ have received significant attention because of the fundamental scientific curiosity in novel physical phenomena arising from intriguing cross-coupling between the dual order parameters^5^, as well as the promise of conceptually novel paradigms for spintronic devices and information storage applications for electrical writing and nondestructive magnetic reading^6^. Although there have been significant efforts in the search for new multiferroic materials in perovskite oxides^7^,^8^,^9^,^10^,^11^,^12^, intrinsic multiferroelectric phases remain largely elusive^13^. The scarcity of such multiferroelectrics is closely related to the violation of the conventional mechanism of cation off-centering for the formation of an electric dipole, which commonly requires an empty *d*^0^ electronic configuration, and the formation of magnetism due to partially filled *d* orbitals. Therefore, a novel design concept and mechanism is highly desired for the search of new multiferroics with conventional ferroelectrics, and this would also have profound implications into the multi-functionalities of perovskite oxides.

Recent revolutionary advances in the field of nanoscale research have opened up fascinating avenues for the exploration of peculiar physical characteristics and novel device concepts^14^,^15^,^16^, and have also driven the quest for unconventional or novel phases in ferroelectrics towards low-dimensional systems^17^,^18^,^19^,^20^. As the size of perovskite ferroelectric oxides approaches the nanometer scale, they suffer from remarkable size and surface effects that manifest themselves in marked deviation of their physical properties from those of their bulk counterparts, such as a decrease in remnant polarization and coercive field, modification of phase transition temperature and domain dynamics^21^,^22^. Discoveries of unconventional phase transitions and associated novel phenomena in finite-dimension ferroelectrics have continued since the early reports of a new low-symmetry ferroelectric ground state^23^ and unconventional antiferroelectric/antiferrodistortion phase transitions^24^,^25^, to more recent new topological phases with exotic polar order such as the toroidal moment and skyrmionic state^26^,^27^. Therefore, phase transitions with nanoscale dimensions emerge as a fertile playground in the pursuit of intriguing physical phenomena and new functional devices paradigms of ferroelectrics.

In this work, we report that PbTiO_3_ nanodots exhibit unusual multiferroic phases with coupled ferroelectricity and ferromagnetism based on first-principles calculations. Nonstoichiometric effects inherent to the nanoscale induce ferromagnetism accompanied by the host ferroelectricity, which could be tailored through engineering of the surface morphology. Furthermore, we predict the key features of the size-dependent phase diagram for the nanodots, which include a rich sequence of ferroelectric-multiferroic-ferromagnetic/nonmagnetic (FE-MF-FM/NM) multiferroic phase transitions. Thus, this work provides a novel pathway to multiferroic transitions in conventional nonmagnetic ferroelectrics.

## Computation Details

All calculations for the PbTiO_3_ nanodots were performed in the framework of density functional theory (DFT) using the generalized gradient approximation corrected with an on-site Hubbard correction *U*^28^, as implemented in the Vienna *ab initio* simulation package (VASP)^29^,^30^. The values of U = 5.0 and J = 0.64 eV were used for Ti 3d states. The projector-augmented wave (PAW) potentials was employed for electron-ion interaction^31^, which explicitly treats the Pb 5*d*, 6*s*, and 6*p*, the Ti 3*s*, 3*p*, 3*d*, and 4*s*, and the O 2s and 2*p* electrons as valence states. The DFT+ *U* scheme provides accurate atomic structures and electronic properties in wide bandgap oxides (i.e., PbTiO_3_) with defects or nonstoichiometry^32^,^33^,^34^. The validity of the method presented here has been further verified by repeating the calculations for small nanowires (Fig. S6) using the more accurate hybrid Hartree-Fock density functional^35^,^36^. The Brillioun-zone integrations were implemented with a 1 × 1 × 1 Monkhorst-Pack *k*-point sampling^37^. The structures and atomic positions were fully relaxed by the conjugate gradient method until the Hellmann-Feynman force acting on each atom is less than 0.01 eV/Å.

The isolated PbTiO_3_ nanodots were constructed using the periodic supercell approach, as shown in Supplementary Fig. S1. A vacuum region of 12 Å was introduced in the [100], [010] and [001] directions, which has been tested large enough to prevent any undesirable interactions between the neighboring nanodots (Fig. S2). Two different nanodots, either terminated by PbO or TiO_2_ atomic facets, were considered to investigate the effect of surface termination. For the morphology of the nanodots, sharp corner (ND-S) and round corner (ND-R) sub-configurations were considered, which were inspired by relevant experimental observations and theoretical implications^38^,^39^,^40^. These nanodots characterize the features of experimentally synthesized nanoparticles^38^,^41^ and act as representatives of nonstoichiometric cases to investigate the intrinsically nonstoichiometric effect on the material properties. The nanodot sizes were characterized by the unit cells arranged along the [100], [010] and [001] directions, which are referred to as *n* × *n* × *n*. The cubic nanodots with dimension *n* varying from 2 to 4 were calculated with a particular focus on the 4 × 4 × 4 type. To determine the ground states of the nanodots, the symmetries of initial structures are broken by displacing Ti atoms along the [001] direction from their center positions or imposing clockwise displacements around [001] axis. We also calculated the several samples of initial configuration with small but random off-center displacement of Ti atoms. We then obtained the ferroelectric ground states by comparing the energies of the resultant structures after full structural relaxations.

## Results and Discussions

**Figure 1:**
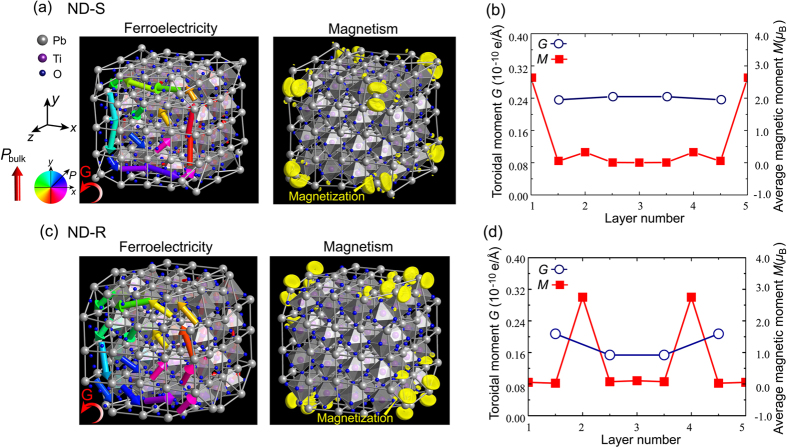
Ferroelectric and ferromagnetic properties of PbO-terminated nanodots, (**a,b**) ND-S and (**c**,**d**) ND-R. The arrows indicate the local polarization in each unit. The contours indicate the angle between the polarization vector **P** and the *x* direction. The yellow areas indicate isosurfaces with magnetic spin densities of 0.02 *μ*_B_/Å^3^. (**b**,**d**) toroidal moment and average magnetic moment in different layers ((*x*-*y*) plane). The integer layer number denotes the Pb-O atomic layer.

**Figure 2:**
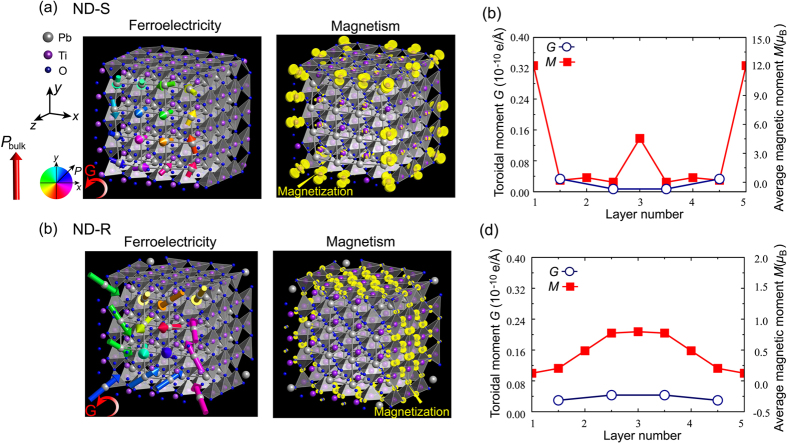
Ferroelectric and ferromagnetic properties of TiO_2_ terminated nanodots, (**a,b**) ND-S and (**c**,**d**) ND-R. The arrows indicate the local polarization in each unit. The contours indicate the angle between the polarization vector **P** and the *x* direction. The yellow areas indicate isosurfaces with magnetic spin densities of 0.02 *μ*_B_/Å^3^. (**b**,**d**) toroidal moment and average magnetic moment in different layers ((*x*-*y*) plane). The integer layer number denotes the Ti-O atomic layer.

The ground state structures of all types of nanodots were found to be *P4/m* symmetry after relaxations. With respect to the ferroelectricity in the nanodots, the local polarization **P**^(i)^ was introduced within each unit cell to analyze the polarization patterns (see Supplementary Information). Figures 1 and 2 depict the vector fields of the electric dipoles in all of the nanodot types (The detailed local polarization in each layer is available in Supplementary Figs S3 and S4). For PbO-terminated ND-S, nontrivial electric dipoles are observed in all of the unit cells, which rotate from cell to cell forming a vortex-like pattern around the [001] axis with negligible net dipole moment. A similar vortex pattern with in-plane curling polarization is also found in the PbO-terminated ND-R (see Fig. 1(b)). The order parameter of the toroidal moment is defined here as 
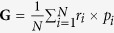
 (*r*_*i*_ denotes a position vector; the sum runs over all the unit cells in the nanodot) to quantify the properties of the ferroelectric vortex. The corresponding values are listed in Table 1 and the layer-by-layer magnitudes of toroidal moments are shown in Fig. 1(b), which signify the existence of vortex polarization in both cases. On the other hand, the polarization configurations in both TiO_2_-terminated cases bear a significant resemblance to the PbO-terminated cases, with the local dipoles curling in the (001) plane characterized by nontrivial toroidal moments, even though the magnitudes of the toroidal moments in these types of nanodots are much smaller than those in the TiO_2_-terminated counterparts (Fig. 2(b)). Thus, ferroelectricity can be sustained in these ultra-small PbTiO_3_ nanodots and manifest in the toroidal ordering with different magnitudes.

The magnetic properties were then investigated by performing total energy calculations of both ferromagnetic and antiferromagnetic configurations. The total magnetic spin moments that are extracted from DFT calculations along with the magnetic charge density (the magnetic moments in per volume) in 4 × 4 × 4 nanodots are summarized in Table 1. The individual magnetic moments of Pb and Ti atoms are listed in the Tables S1 to S3. It can be seen all types of nanodots spontaneously develop ferromagnetic ground states with emerging magnetic moments despite the nonmagnetic nature of bulk PbTiO3 crystal. The PbO-terminated ND-S exhibits a nontrivial total magnetic moment of 6.00 *μ*_*B*_ (0.82 *μ*_*B*_/nm^3^) and the magnetic moments are in the ferromagnetic alignment since the energy difference between these orders ΔE = E_tot_^FM^ − E_tot_^AFM^ is negative (−0.29 meV). The same moments arranged in ferromagnetic configuration (ΔE = −0.97 meV) persist when the apexes of the nanodot are removed. Similar to the scenarios for the PbO-terminated nanodots, two types of TiO_2_-terminated nanodots are also found to be ferromagnetic. The ND-S displays a giant magnetic moment of 28.00 *μ*_*B*_ (3.85 *μ*_*B*_/nm^3^), while these magnetic moments decrease markedly in magnitude 3.96 *μ*_*B*_ (0.54 *μ*_*B*_/nm^3^) in ND-R. Therefore, the ferromagnetism is intrinsic to the PbTiO_3_ nanodots, regardless of the lateral surface terminations and edge characteristics, and coexists with the host ferroelectricity, which enables the 4 × 4 ×4  dots to act as zero-dimensional multiferroic nanoparticles. Note that the calculations of the directions of the magnetic moments in current large systems are not computationally feasible at present by noncollinear magnetic calculations. However, their directions may partly be related to the structure symmetries and resulting polar directions, which potentially suggest magnetoelectric coupling.

Having demonstrated the coexistence of ferroelectricity and magnetism, we proceeded to analyze the multiferroic pattern in these nanodots. In the PbO-terminated nanodots (see Fig. 1 and Supplementary Fig. S3), ferroelectricity exists in all of the unit cells, whereas the spin-densities originate entirely from low-coordination Pb atom sites; the moments in the ND-S are localized near eight apex Pb atoms, while those in the ND-R are concentrated at the intersection of the (100) and (010) planes (edge area), which results in a multiferroic vertex/edge. For TiO_2_-terminated ND-S (see Fig. 2(a)), the induced spin moments are mainly delocalized on the unsaturated apex and edge Ti atoms, with little contribution from the surface or interior Ti atoms, which gives rise to a mixed multiferroic-ferroelectric phase state in this nanosized system. Note that the emerging magnetic moments transfer to the O atoms located on the lateral facets ([100] and [010] surfaces) as soon as the apexes are removed, as illustrated in Fig. 2(b). The TiO_2_-terminated ND-R thus forms a nanocomposite in the manner of a multiferroic surface combined with a ferroelectric core. These termination-dependent multiferroic characteristics provide a prospective means of tailoring multiferroic patterns with special functionalities at the nanoscale through morphology engineering.

**Figure 3:**
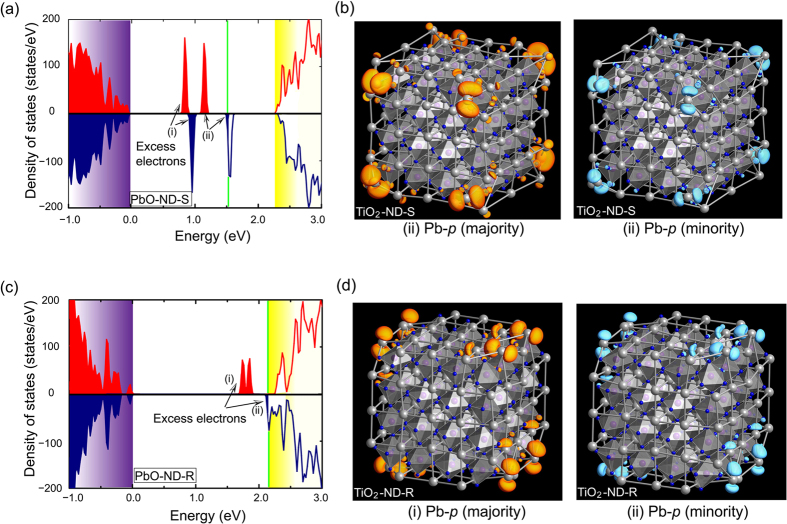
Electronic DOS for the PbO-terminated nanodots (**a**) ND-S and (**c**) ND-R. The highest occupation level of oxygen 2*p* is set as the zero energy. The green lines denote the Fermi level, which is set at 0.0 eV. The red and blue lines indicate the majority- and minority-spin orbitals, respectively. (**b**) Squared wave functions of the spin-polarized in-gap states (ii) in PbO-terminated ND-S. (**d**) Squared wave functions of in-gap states (i) and (ii) in PbO-terminated ND-R. The orange and light-blue colors indicate isosurfaces of majority- and minority-spin densities of 0.001 Å^−3^, respectively.

**Figure 4:**
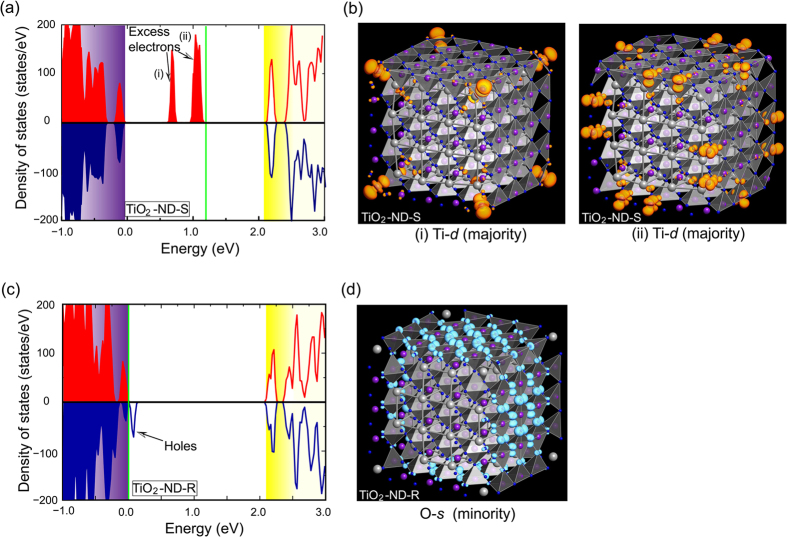
Electronic DOS for the TiO_2_-terminated nanodots, (**a**) ND-S and (**c**) ND-R. The highest occupation level of oxygen 2*p* is set as the zero energy. The green lines denote the Fermi level, which is set at 0.0 eV. The red and blue lines indicate the majority- and minority-spin orbitals, respectively. (**b**) Squared wave functions of in-gap states (i) and (ii) in TiO_2_-terminated ND-R. (**d**) Squared wave functions of the in-gap states in TiO_2_-terminated ND-S. The orange and light-blue colors indicate isosurfaces of majority- and minority-spin densities of 0.001 Å^−3^, respectively.

To clarify the underlying mechanism for the emerging magnetic moments and their unusual configurations, the full electronic density of states (DOS) of each nanodot were analyzed, as shown in Figs 3 and 4. In the case of magnetic PbO-terminated ND-S, four new isolated states can be observed within the bandgap, which are absent in bulk PbTiO_3_ crystal. The two lower intragap states (i) are occupied by equal majority- and minority-spin electrons in a spin-unpolarized state (see also Supplementary Fig. S5), while the upper pair (ii) is spin-polarized as the minority spin crosses the Fermi level containing a smaller portion of occupied electrons with respect to the majority spin. These spin-polarized electron states thus contribute to the net magnetism in the dot. The spin-polarized excess electrons originate from the nonstoichiometry of the nanodot; the atomic composition of the PbO-terminated ND-S is comprised of (*n* + 1)^3^ Pb, *n*^3^ Ti and 3*n*^2^ (*n* + 1) O atoms, with a deviation from the stoichiometric composition with a 1:1:3 ratio of the three atoms in bulk PbTiO_3_. This nonstoichiometric composition results in a structure with a net charge of 6*n* + 2 (*n* = 4 in this case) compared to the nominal ionic charges of Pb^2+^, Ti^4+^, and O^2−^, which yields a charge imbalance and heavy *n*-type electronic character. The squared wave functions shown in Fig. 3(b) indicate that these spin-polarized states are derived from the *p* orbitals of Pb atoms. A similar *n*-type character and net spin density of the occupied levels are also apparent in the ND-R, as illustrated in Fig. 3(c). The spin-polarized charge density, however, predominantly appears around the edge atoms due to the dangling bonds of the Pb atoms (Fig. 3(d)), in line with the distribution of emerging magnetism.

The spin-resolved DOS of TiO_2_-terminated nanodots were also investigated and the results are pictured in Fig. 4, in which the two types of nanodots exhibit distinct electronic characteristics with either excess electrons or holes due to their respective nonstoichiometric compositions. As clearly shown in Fig. 4(a) for the ND-S, two distinct electronic states appear below the Fermi energy. Each state is partially occupied by majority-spin electrons; therefore, these states are also spin-polarized and give rise to a net magnetic moment in the nanodot. The excess electrons are attributable primarily to the *d* orbital of under-coordinated Ti atoms from inspection of the squared wave function shown in Fig. 4(b), which is different from those for the PbO-terminated nanodots. In contrast to the case for ND-S, the DOS for ND-R indicates typical *p*-type characteristics, as illustrated in Fig. 4(c). The spin-down orbital crosses the Fermi energy and has a metallic nature, while the spin-up channel is insulating with a bandgap, which is indicative of half metallic behavior. The asymmetric state densities between the majority and minority signify that the DOS is also spin-polarized, and thereby accounts for the unexpected ferromagnetism in TiO_2_-terminated ND-R. Based on the same analyses of the overall composition and net valence state, the charge imbalance is also identified in this type of nanodot, which introduces four holes into the system. These electronic holes reside on the surface O atom with *p* orbital characteristics, which is consistent with the position of the emerging magnetic moments. Thus, it is the free carriers (electrons or holes) contributed from the nanometer-sized nonstoichiometric effects in the nanodots that cause magnetism with spatially delocalized spin moments and that fundamentally determine the pattern of the multiferroic/ferroelectric nanocomposite. It is worth to mention that these considered nanodots, with the exception of TiO_2_-terminated ND-S that is entirely insulator, possess half metallic behaviors either at the edge or on the surface.

Finally, the magnitude of the magnetic moments and electric orders in nanodots with different diameters were investigated to characterize the size-dependent multiferroic behavior. Table 1 lists the calculated multiferroic features for the various types of nanodots. It is apparent that magnetism (magnetic moment density) is progressively enhanced as the nanodot size is reduced, which is strongly correlated with the larger surface-to-bulk ratio and the increasingly pronounced nonstoichiometric effects. In contrast, the toroidal moment generally decreases with a decrease in the nanodot diameter, which could be associated with poor screening of the depolarization field. In particular, the ferroic order in TiO_2_-terminated ND-S disappears as the size decreases to 2 × 2 ×2 . As a result, the nanodot becomes pure ferromagnetic and the MF-FM phase transition is triggered at a critical size of approximately 1.0 nm in diameter. Note that the ferroelectric dipole is a collective phenomenon arising from the interaction between long-range Coulomb interactions that favor ferroelectric distortion and short-range interactions that stabilize centrosymmetric structures^42^. The critical size for ferroelectricity in the nanodots is consistent with the predicted minimum effective range of the coulombic force, which is a few unit cells in size^43^, beyond which the collective ferroelectric distortion can be stabilized. On the other hand, the magnetic moments tend to diminish continuously with an increase in particle size (Supplementary Fig. S6) because the nonstoichiometric effect is weakened with the increasing volume of the nanodots, in which the free carriers and volume are increased by factors of *n* and *n*^3^, respectively. Therefore, the number of spin-polarized free carriers per unit volume and the resultant spin density are virtually negligible in extremely large nanodots, triggering an MF-FE phase transition. Therefore, PbTiO_3_ nanodots undergo rich multiferroic phase transitions associated with the particle dimensions, which go beyond the well-known ferroelectric-paraelectric transformation with increased temperature or pressure^44^,^45^.

The present results thus suggest a novel strategy for low-dimensional multiferroics by engineering the nonstoichiometric effects in PbTiO_3_ nanodots. On experiments, ferroelectric nanoparticles with diameter of 3–10 nanometers have been manufactured and smaller particles are anticipated in the near future due to recent advances in the bottom-up nano-manufacturing techniques^46^,^49^. Besides, the edge structure is the typical characteristics of perovskite oxides nanostructures^39^,^50^,^51^, while ferroelectric nanodots with similar round corner morphology have also be fabricated experimentally^38^. It is also worth to mention that the vortex-type polarization has been observed directly in low-dimensional systems^52^,^53^. Thus, the characteristics of the models are realistic and it is highly possible to realize low-dimensional multiferroics experimentally as proposed here. Moreover, the predicted multiferroic phase transitions raised by the pronounced nanometer-sized nonstoichiometric effects are not unique to these nanodots but can also be extrapolated to other finite dimensional nanocrystalline forms, such as nanowires and nanorods, due to their similar compositional deficiency. Therefore, the present work may boost further theoretical and experimental investigations on various low-dimensional functional oxides to explore novel physical phenomena and functionalities.

In summary, we have presented unconventional multiferroic phases with emerging magnetism and magnetoelectric coupling in ferroelectric PbTiO_3_ nanodots by first-principles calculations. The intrinsic nanometer-sized nonstoichiometric effects induce ferromagnetism in coexistence with the host ferroelectric orders. The multiferroic order is susceptible to surface termination and morphology, which enables custom tailoring of the properties at the nanoscale. The size dependence of the multiferroic properties was investigated and possible FE-MF-FM/NM phase transitions were predicted. The present results thus open up exciting prospects for low-dimensional multiferroics and multifunctional oxides that exhibit magnetism that coexists with the host functionalities by nonstoichiometric engineering in nonmagnetic finite-dimensional systems.

## Additional Information

**How to cite this article:** Xu, T. *et al*. Multiferroic Phases and Transitions in Ferroelectric Lead Titanate Nanodots. *Sci. Rep.* **7**, 45373; doi: 10.1038/srep45373 (2017).

**Publisher's note:** Springer Nature remains neutral with regard to jurisdictional claims in published maps and institutional affiliations.

## Supplementary Material

Supplementary Information

## Figures and Tables

**Table 1:** Multiferroic properties, i.e., toroidal moment |**G**| (10^−10^ e/Å) about the [001] axis, magnetic moment *M (μ*_*B*_), and magnetic moment density *M*/Ω (*μ*_*B*_/nm^3^), in various nanodots with different surface morphologies and diameters.

Termination	Edge character	Size	Ferroelectricity	Magnetism		Ferroic phase
			G (10^−10^ e/Å)	*M (μ*_*B*_)	*M*/Ω(*μ*_*B*_/nm^3^)	
PbO	ND-S	**4 × 4 × 4**	**0.24**	**6.00**	**0.82**	**MF**
		3 × 3 × 3	0.19	4.00	1.07	MF
		2 × 2 × 2	0.00	0.00	0.00	NM
					
	ND-R	**4 × 4 × 4**	**0.18**	**6.00**	**0.83**	**MF**
		3 × 3 × 3	0.20	0.00	0.00	FE
		2 × 2 × 2	0.10	2.00	1.28	MF
TiO_2_	ND-S	**4 × 4 × 4**	**0.02**	**28.00**	**3.85**	**MF**
		3 × 3 × 3	0.01	22.00	5.96	MF
		2 × 2 × 2	0.00	16.00	10.18	FM
					
	ND-R	**4 × 4 × 4**	**0.03**	**3.96**	**0.54**	**MF**
		3 × 3 × 3	0.00	6.08	1.62	FM
		2 × 2 × 2	0.00	0.00	0.00	NM
