# Supplementary material for: Multiferroic Phases and Transitions in Ferroelectric Lead Titanate Nanodots
Source: Sci Rep. 2017 Apr 3;7:45373. doi: 10.1038/srep45373 (PMC5377253; doi:10.1038/srep45373)
Supplement: Supplementary Information [file srep45373-s1.pdf]

Supporting Information

Title: Multiferroic Phases and Transitions in Ferroelectric Lead Titanate Nanodots

Tao Xu,[1,*] Takahiro Shimada,[1,**] Yoshitaka Uratani,[1] Xiaoyuan Wang,[2] Jie Wang,[3] and Takayuki Kitamura[1]

[1] *Department of Mechanical Engineering and Science, Kyoto University, Nishikyo-ku, Kyoto 615-8540, Japan*

[2] *Institute of Systems Engineering, China Academy of Engineering Physics, Postbox 919-401, Mianyang 621900, China*

[3] *Department of Engineering Mechanics, School of Aeronautics and Astronautics, Zhejiang University, Hangzhou 310027, China*

Definition of local polarization.

The ferroelectric properties in the nanodots are analyzed by introducing the site-specific local polarization p_i as:

$$p_i = \frac{e}{\Omega_c} \sum_j w_j \mathbf{Z}_j^* \mathbf{u}_j \,, \tag{1}$$

where Ω_c is the volume of the local unit cell i; e and $\mathbf{u_j}$ denote the electron charge and the atomic displacement vector relative to the ideal lattice site of atom j, respectively. The index j covers all atoms in the local unit cell i. $\mathbf{Z}_j^*$ is the Born effective charge tensor of atom j and w_j is a weight factor, determined by the number of unit cells that share atom j. The local polarization are

T.Xu* and T.Shimada** contributed equally to this work.
* E-mail: xu.tao.44a@st.kyoto-u.ac.jp
** E-mail: shimada@me.kyoto-u.ac.jp

evaluated for Ti (PbO-terminated NDs) and Pb (TiO_2-terminated NDs) centered unit cells, respectively. The weight w_j , is set as $w_{\text{Pb}} = 1$, $w_{\text{Ti}} = 1/8$, and $w_{\text{O}} = 1/2$ for the Pb-centered local unit cells , and $w_{\text{Pb}} = 1/8$, $w_{\text{Ti}} = 1$, and $w_{\text{O}} = 1/2$ for the Ti-centered local unit cells.

References

1. Blochl, P. E. Projector augmented-wave method. *Phys. Rev. B* **50**, 17953 (1994).

2. Kresse, G.; Hafner, J. Ab initio molecular dynamics for liquid metals. *Phys. Rev. B* **47**, 558 (1993).

3. Kresse, G.; Furthmüller, J. Efficient iterative schemes for ab initio total-energy calculations using a plane-wave basis set. *Phys. Rev. B* **54**, 11169 (1996).

4. Anisimov, V. I.; Aryasetiawan, F. and Liechtenstein, A. I. First-principles calculations of the electronic structure and spectra of strongly correlated systems: the LDA + U method. *J. Phys.: Condens. Matter* **9**, 767-808 (1997).

5. Monkhorst, H. J.; Pack, J. D. Special points for Brillouin-zone integrations. *Phys. Rev. B* **13**, 5188 (1976).

**Layre1**	1	2	3	4	5
1	0.433	0.077	0.002	0.058	0.433
2	0.058	0.005	0.001	0.005	0.077
3	0.002	0.001	0.000	0.001	0.002
4	0.077	0.005	0.001	0.005	0.058
5	0.433	0.058	0.002	0.077	0.433
**Layer2**	1	2	3	4	5
1	0.069	0.002	0.001	0.000	0.069
2	0.000	0.000	0.000	0.000	0.002
3	0.001	0.000	0.000	0.000	0.001
4	0.002	0.000	0.000	0.000	0.000
5	0.069	0.000	0.001	0.002	0.069
**Layer3**	1	2	3	4	5
1	0.001	0.000	0.000	0.000	0.001
2	0.000	0.000	0.000	0.000	0.000
3	0.000	0.000	0.000	0.000	0.000
4	0.000	0.000	0.000	0.000	0.000
5	0.001	0.000	0.000	0.000	0.001
**Layer4**	1	2	3	4	5
1	0.069	0.002	0.001	0.000	0.069
2	0.000	0.000	0.000	0.000	0.002
3	0.001	0.000	0.000	0.000	0.001
4	0.002	0.000	0.000	0.000	0.000
5	0.069	0.000	0.001	0.002	0.069
**Layer5**	1	2	3	4	5
1	0.433	0.077	0.002	0.058	0.433
2	0.058	0.005	0.001	0.005	0.077
3	0.002	0.001	0.000	0.001	0.002
4	0.077	0.005	0.001	0.005	0.058
5	0.433	0.058	0.002	0.077	0.433

Supplementary Table S1. The individual magnetic moments of the Pb atoms in each layer (x-y plane) of the PbO-terminated ND-S. The row and column numbers is the position of Pb atom in x and y directions, respectively.

**Layre1**	1	2	3	4	5
1		0.008	0.003	0.001	
2	0.001	0.001	0.000	0.001	0.008
3	0.003	0.000	0.000	0.000	0.003
4	0.008	0.001	0.000	0.001	0.001
5		0.001	0.003	0.008	
**Layer2**	1	2	3	4	5
1	0.442	0.136	0.005	0.004	0.442
2	0.004	0.000	0.002	0.000	0.136
3	0.005	0.002	0.000	0.002	0.005
4	0.136	0.000	0.002	0.000	0.004
5	0.442	0.004	0.005	0.136	0.442
**Layer3**	1	2	3	4	5
1	0.023	0.000	0.002	-0.001	0.023
2	-0.001	0.000	0.000	0.000	0.000
3	0.002	0.000	0.000	0.000	0.002
4	0.000	0.000	0.000	0.000	-0.001
5	0.023	-0.001	0.002	0.000	0.023
**Layer4**	1	2	3	4	5
1	0.442	0.136	0.005	0.004	0.442
2	0.004	0.000	0.002	0.000	0.136
3	0.005	0.002	0.000	0.002	0.005
4	0.136	0.000	0.002	0.000	0.004
5	0.442	0.004	0.005	0.136	0.442
**Layer5**	1	2	3	4	5
1		0.008	0.003	0.001	
2	0.001	0.001	0.000	0.001	0.008
3	0.003	0.000	0.000	0.000	0.003
4	0.008	0.001	0.000	0.001	0.001
5		0.001	0.003	0.008	

Supplementary Table S2. The individual magnetic moments of the Pb atoms in each layer (x-y plane) of the PbO-terminated ND-R. The row and column numbers is the position of Pb atom in x and y directions, respectively.

**Layre1**	1	2	3	4	5
1	0.902	1.006	1.020	0.001	0.902
2	0.001	0.050	0.042	0.050	1.006
3	1.020	0.042	0.004	0.042	1.020
4	1.006	0.050	0.042	0.050	0.001
5	0.902	0.001	1.020	1.006	0.902
**Layer2**	1	2	3	4	5
1	0.001	0.050	0.042	0.002	0.001
2	0.002	0.010	0.012	0.010	0.050
3	0.042	0.012	0.005	0.012	0.042
4	0.050	0.010	0.012	0.010	0.002
5	0.001	0.002	0.042	0.050	0.001
**Layer3**	1	2	3	4	5
1	1.022	0.042	0.004	0.047	1.022
2	0.047	0.014	0.006	0.014	0.042
3	0.004	0.006	0.005	0.006	0.004
4	0.042	0.014	0.006	0.014	0.047
5	1.022	0.047	0.004	0.042	1.022
**Layer4**	1	2	3	4	5
1	0.001	0.050	0.042	0.002	0.001
2	0.002	0.010	0.012	0.010	0.050
3	0.042	0.012	0.005	0.012	0.042
4	0.050	0.010	0.012	0.010	0.002
5	0.001	0.002	0.042	0.050	0.001
**Layer5**	1	2	3	4	5
1	0.902	1.006	1.020	0.001	0.902
2	0.001	0.050	0.042	0.050	1.006
3	1.020	0.042	0.004	0.042	1.020
4	1.006	0.050	0.042	0.050	0.001
5	0.902	0.001	1.020	1.006	0.902

Supplementary Table S3. The individual magnetic moments of the Ti atoms in each layer (*x*-*y* plane) of the TiO_2-terminated ND-S. The row and column numbers is the position of Ti atom in *x* and *y* directions, respectively.

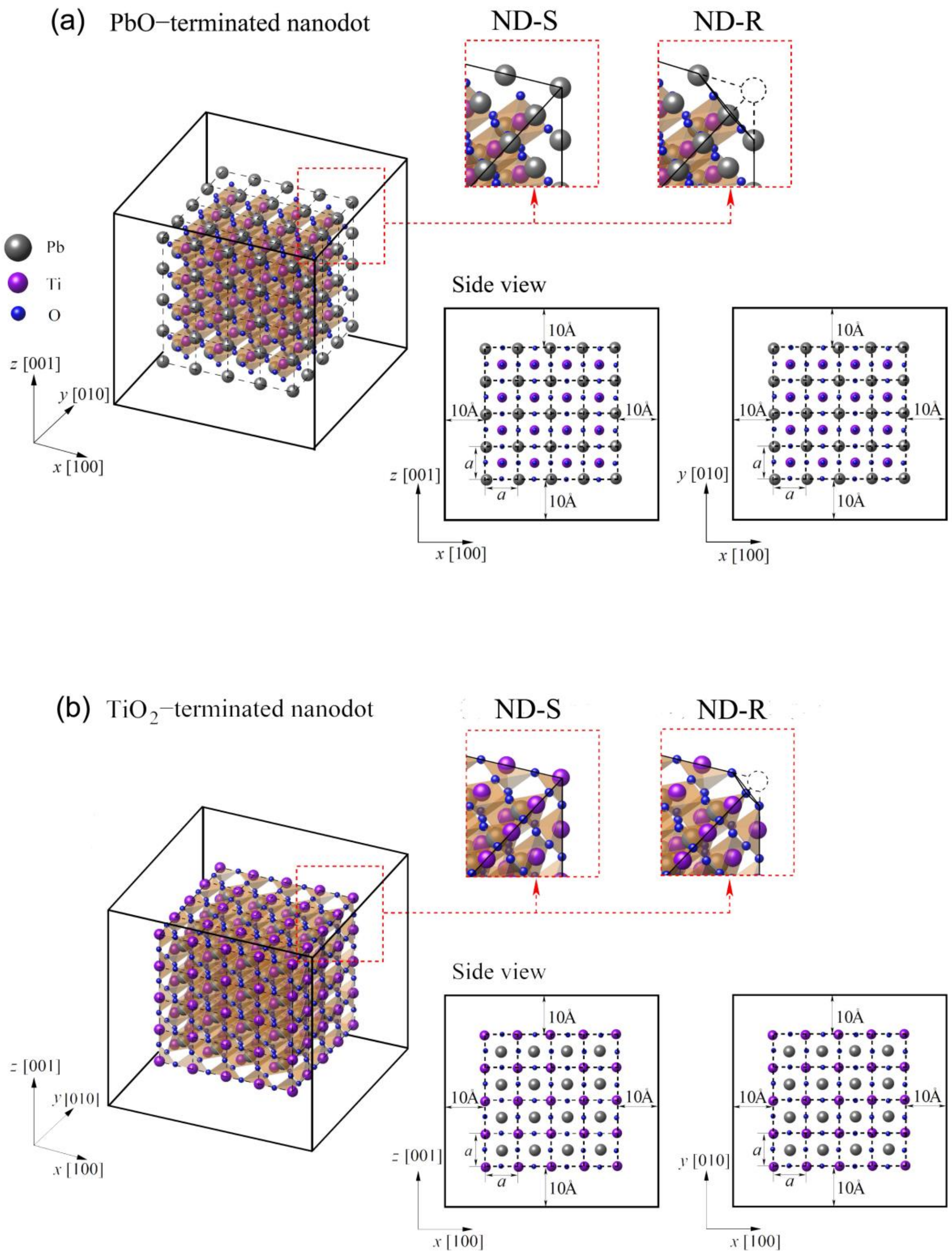

Supplementary Figure S1 Simulation models of $PbTiO_3$ nanodots with (a) PbO and (b) TiO_2 termination. Each configuration is divided into sharp corner (ND-S) and round corner (ND-R). In the ND-R, the apex Pb or Ti atoms are romoved.

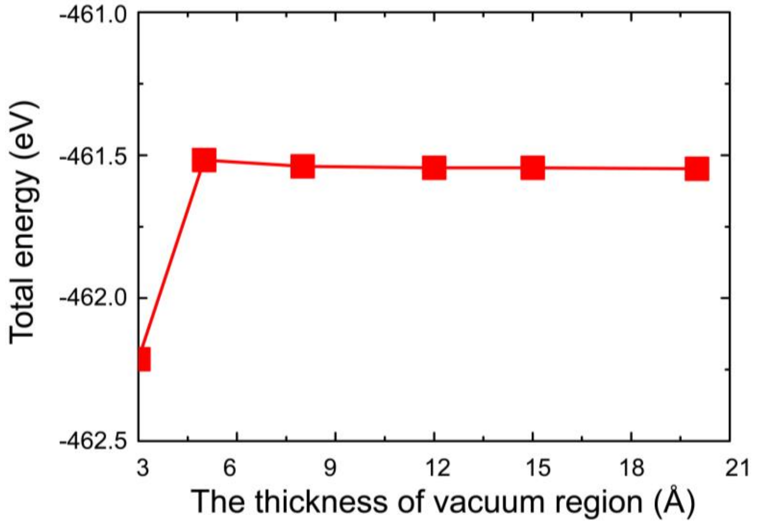

Supplementary Figure S2 Total energy for the simulation supercell as a function of vacuum region thickness. The total energy sufficiently converges when the vacuum thickness is equal to 12 Å.

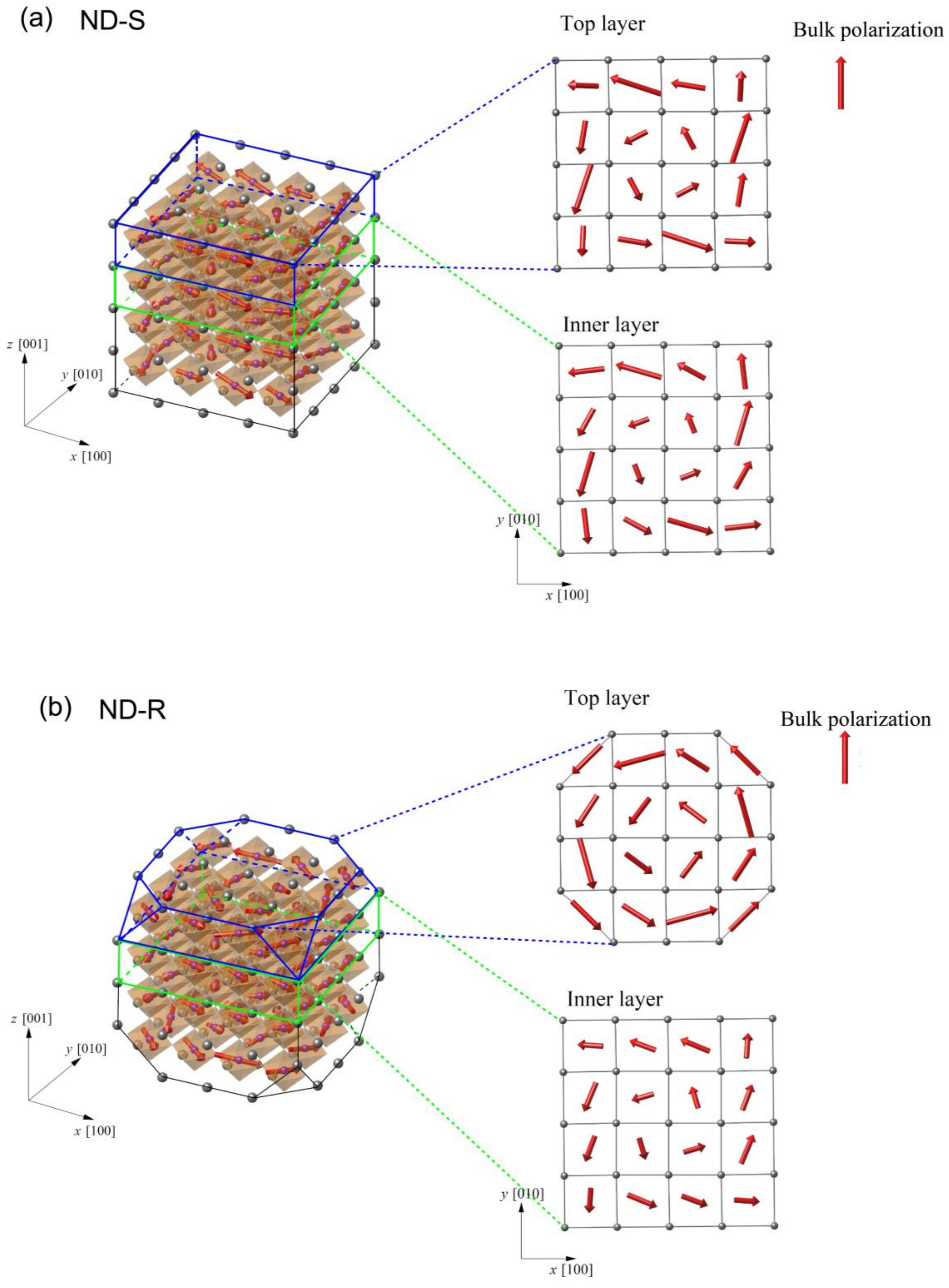

Supplementary Figure S3 Distribution of local polarization in the top and inner layers of PbO-terminated nanodots.

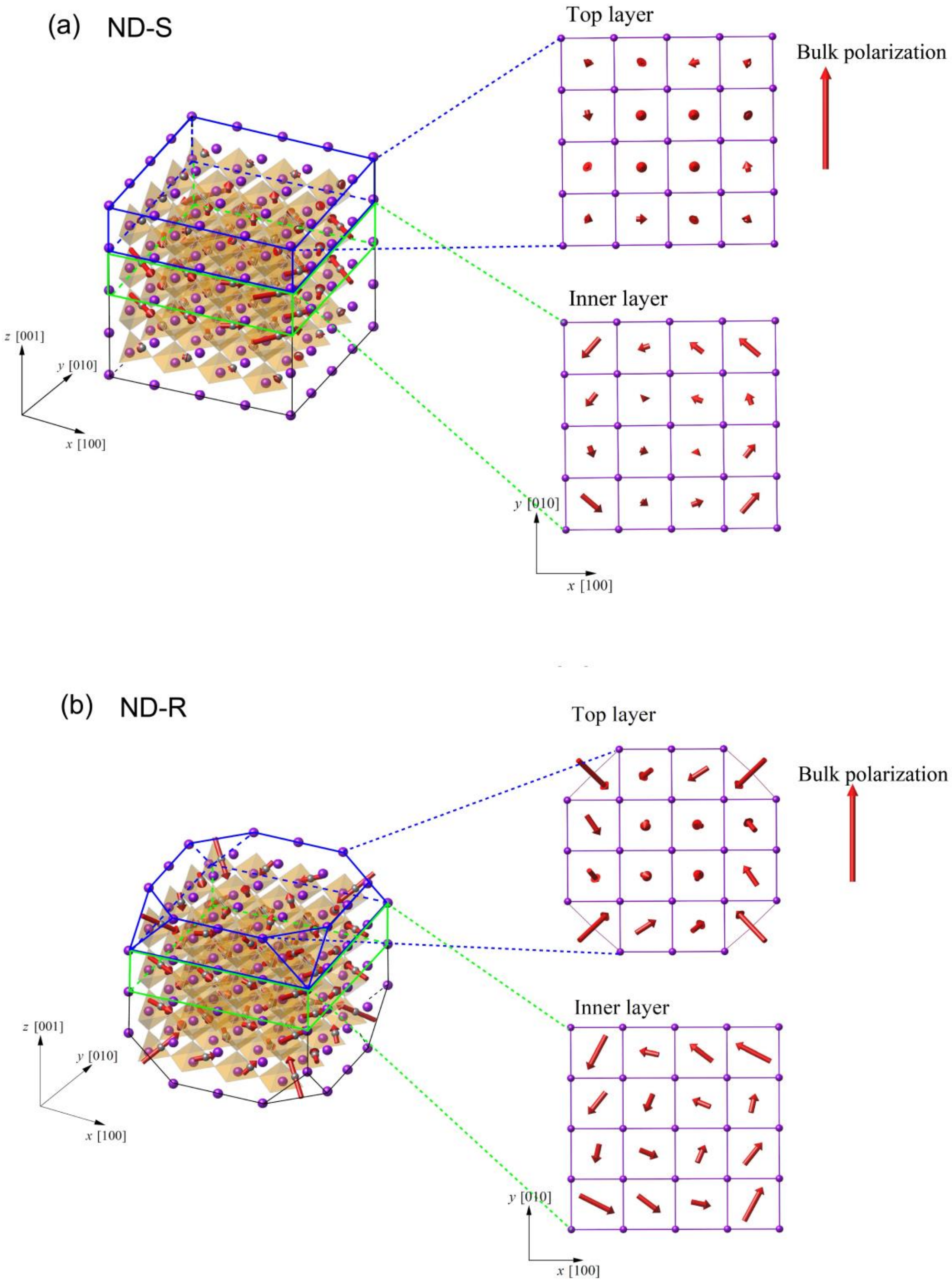

Supplementary Figure S4 Distribution of local polarization in the top and inner layers of TiO_2-terminated nanodots.

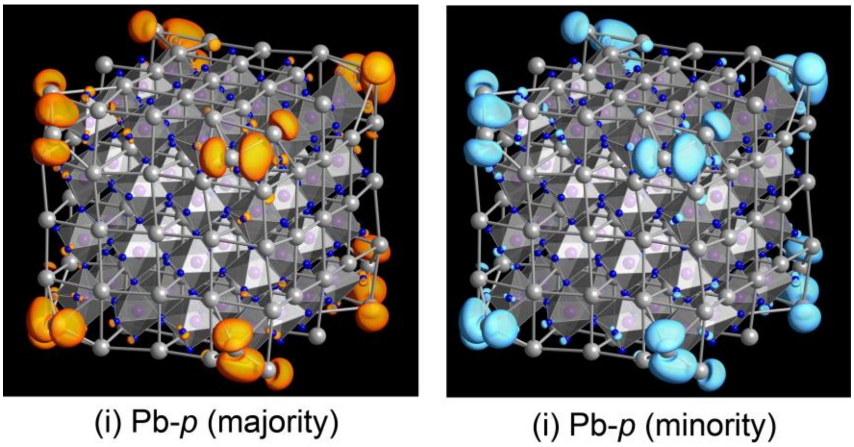

Supplementary Figure S5 The squared wave functions of lower intragap states (i) of PbO-terminated ND-S.

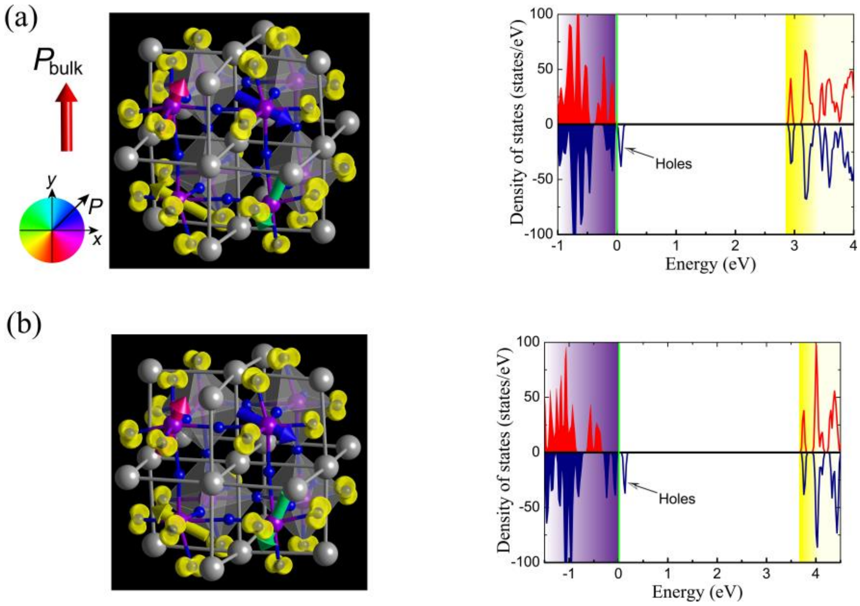

Supplementary Figure S6 The results of multiferroic and electronic properties of PbO-terminated ND-R using (a) DFT+U (b) HSE method for PbO-terminated ND-R. The total magnetic moments are 2 μ_B in both cases.
